# International Society of Sports Nutrition Position Stand: beta-hydroxy-beta-methylbutyrate (HMB)

**DOI:** 10.1186/1550-2783-10-6

**Published:** 2013-02-02

**Authors:** Jacob M Wilson, Peter J Fitschen, Bill Campbell, Gabriel J Wilson, Nelo Zanchi, Lem Taylor, Colin Wilborn, Douglas S Kalman, Jeffrey R Stout, Jay R Hoffman, Tim N Ziegenfuss, Hector L Lopez, Richard B Kreider, Abbie E Smith-Ryan, Jose Antonio

**Affiliations:** 1 Department of Health Sciences and Human Performance, University of Tampa, Tampa, FL, USA; 2 Division of Nutritional Sciences, University of Illinois, Urbana, IL, USA; 3 Exercise and Performance Nutrition Laboratory, Dept. of Physical Education and Exercise Science, University of South Florida, 4202 E. Fowler Avenue, PED 214, Tampa, FL, 33620, USA; 4 Department of Nutritional Sciences, Rutgers, The State University of New Jersey, New Brunswick, NJ, USA; 5 Laboratory of Applied Nutrition and Metabolism, Physical Education and School of Sports, University of São Paulo, São Paulo, Brazil; 6 Human Performance Laboratory, Exercise & Sport Science Department, University of Mary Hardin-Baylor, Belton, TX, 76513, USA; 7 Miami Research Associates, Endocrinology & Nutrition Department, 6141 Sunset Drive - Suite 301, Miami, FL, 33143, USA; 8 Institute of Exercise Physiology and Wellness, University of Central Florida, Orlando, FL, 32816, USA; 9 The Center for Applied Health Sciences, Stow, OH, 44224, USA; 10 Supplement Safety Solutions, Bedford, MA, USA; 11 Exercise & Sport Nutrition Lab, Department of Health & Kinesiology, Texas A&M University, College Station, TX, USA; 12 Applied Physiology Laboratory, Department of Exercise and Sport Science, University of North Carolina Chapel Hill, Chapel Hill, NC, 27599-8605, USA; 13 Exercise and Sports Science, Nova Southeastern University, Davie, FL, 33314, USA

## Abstract

Position Statement: The International Society of Sports Nutrition (ISSN) bases the following position stand on a critical analysis of the literature on the use of beta-hydroxy-beta-methylbutyrate (HMB) as a nutritional supplement. The ISSN has concluded the following. 1. HMB can be used to enhance recovery by attenuating exercise induced skeletal muscle damage in trained and untrained populations. 2. If consuming HMB, an athlete will benefit from consuming the supplement in close proximity to their workout. 3. HMB appears to be most effective when consumed for 2 weeks prior to an exercise bout. 4. Thirty-eight mg·kg·BM^-1^ daily of HMB has been demonstrated to enhance skeletal muscle hypertrophy, strength, and power in untrained and trained populations when the appropriate exercise prescription is utilized. 5. Currently, two forms of HMB have been used: Calcium HMB (HMB-Ca) and a free acid form of HMB (HMB-FA). HMB-FA may increase plasma absorption and retention of HMB to a greater extent than HMB-CA. However, research with HMB-FA is in its infancy, and there is not enough research to support whether one form is superior. 6. HMB has been demonstrated to increase LBM and functionality in elderly, sedentary populations. 7. HMB ingestion in conjunction with a structured exercise program may result in greater declines in fat mass (FM). 8. HMB’s mechanisms of action include an inhibition and increase of proteolysis and protein synthesis, respectively. 9. Chronic consumption of HMB is safe in both young and old populations.

## Introduction

Supplementing the diet with the amino acid leucine in combination with resistance training may increase lean body mass (LBM), strength and decrease body fat [1-3]. Moreover, leucine appears to decrease skeletal muscle soreness following eccentric exercise [4], and prevent declines in both circulating testosterone and skeletal muscle power following an overreaching cycle [5]. Leucine has been thought to augment adaptations to strength training by acting as the primary signal to activate protein synthesis (e.g. regulation of translation initiation) [1]. Additionally, for over three decades this amino acid has been known to exert antiproteolytic effects [6]. However, the effects of leucine on muscle proteolysis are maximized at 10–20 times (5–10 mM·L^−1^) the concentration required to maximally stimulate muscle protein synthesis [6]. Thus, it is probable that these effects are partly mediated by the conversion of leucine to a specific metabolite [7]. One strong candidate is the leucine-derived metabolite, beta-hydroxy-beta-methylbutyrate (HMB) [7,8]. In 1996, Nissen et al. [7] first demonstrated that supplementation with HMB lowered muscle proteolysis following resistance training, and augmented gains in LBM and strength in a dose-dependent manner. Since that time HMB has been studied in a variety of anaerobic and aerobic training conditions ([9]). While numerous studies have supported the efficacy of HMB supplementation for enhancing recovery [10,11], LBM [10,12], strength [7], power [13], and aerobic performance [14], there have been conflicting results (Tables 1 and 2). For this reason, the primary purpose of this Position Stand is to critically analyze the existing literature on HMB supplementation and provide careful recommendations on how to optimize its effects on body composition, strength, power, and aerobic performance across varying levels of age, sex, and training status. The second purpose of this Position Stand is to critically discuss the current and proposed mechanisms of action of HMB.

**Table 1:** HMB effects on indices of skeletal muscle damage and breakdown

**Experiment**	**Subjects**	**Protocol**	**Diet control**	**Duration**/**dose**	**Additional supplements**	**Timing**	**Damage indices**	**Outcome**
Nissen 1996 [7]	Untrained, college-aged males	Progressive Free Weights	Yes	3 weeks, 1.5 or 3 grams per day HMB-Ca	No	1 gram with each of 3 meals, No timing relative to training	CK, LDH, 3-MH	With HMB-Ca CK, LDH, and 3-MH all decreased in a dose dependent manner with 20–60 % declines in CK and LDH and 20 % declines in 3-MH, the marker of protein breakdown
Jowko 2001 [10]	Active, college-aged males	Progressive Free Weights	No	3 weeks, 3 grams per day HMB-Ca	20 grams creatine per day for 7 days followed by 10 grams per day for 14 days	1 gram with each of 3 meals, No timing relative to training	CK and Urine and Plasma Urea	26-46 % decrease in serum and urine urea nitrogen with HMB-Ca and HMB-Ca lowered CK by 189 %
Kreider 1999 [15]	NCAA Football Players	Instructed to not change current training Regimen	No	28 days, 3 grams per day HMB-Ca	No	1 gram with each of 3 meals, No timing relative to training	CK	No Effect
Paddon-Jones 2001 [16]	Untrained college-aged males	1 isokinetic bout of exercise for elbow flexors	No	6 days prior to bout, 3 grams per day HMB-Ca	No	1 gram with each of 3 meals, No timing relative to training	CK, Soreness, Arm girth, Strength	No Effect
Wilson 2009 [17]	Untrained college-aged males	1 isokinetic, eccentric bout for knee extensors and flexors	Yes	3 grams HMB-Ca	No	60 minutes pre vs. Immediately post exercise	CK, LDH, Soreness	Pre Exercise HMB-Ca: Prevented the rise in LDH and tended to decrease soreness. Post exercise HMB-Ca, No effects suggesting a possible effect of dosage timing on outcomes.
Kreider 2000 [18]	NCAA Football Players	Offseason Strength and Conditioning Program	No	3 grams HMB-Ca	No	1 gram with each of 3 meals, No timing relative to training	CK, LDH	No Effect
Knitter 2000 [11]	Trained runners 20–50 yrs of age who ran a minimum of , 48 km per week	20 km run	No	6 weeks, 3 grams per day HMB-Ca	No	1 gram with each of 3 meals, No timing relative to training	CK	HMB-Ca decreased serum CK by approximately 50 %
Hoffman 2004 [19]	NCAA Football players	Football camp	No	10 days, 3 grams per day HMB-Ca	No	1 gram with each of 3 meals, No timing relative to training	CK, soreness	No Effect
Panton et al. 2000 [20]	Men and women, divided into untrained and resistance trained (> 6 months), 20–40 yrs of age	Monitored 4 wk high intensity progressive resistance training	No	4 weeks, 3 grams per day HMB-Ca	No	1 gram with each of 3 meals, No timing relative to training	CK	CK increased 16 and 46 % in men and women, respectively, in the placebo group. In the HMB group CK increased by 3 % and decreased by 12 % in men and women, respectively
Van Someran 2005 [21]	Untrained college-aged males	Eccentric bout of free weight exercise for elbow flexors	No	14 days, 3 grams per day	0.3 g alpha-ketoisocaproic acid per day	1 gram with each of 3 meals, No timing relative to training	CK, Soreness	Completely prevented exercise induced rise in CK, and blunted the increase in soreness

**Table 2:** HMB effects on body composition and performance^*^

**Experiment**	**Subjects**	**Protocol**	**Periodized**	**Diet control**	**Duration**/**dose**	**Additional supplements**	**Body composition measures**	**Performance measures**	**Outcomes of HMB**-**Ca supplementation relative to placebo**
Nissen 1996 [7]	Trained, NCAA football players	Monitored progressive resistance training	No	No	7 weeks, 3 grams per day HMB-Ca	No	TOBEC for total FFM and FM	Bench Press and Squat	FFM: + 1.9 % FM: - 0.5 % Strength: + 2.3 % average
Nissen 1996 [7]	Untrained college-aged males	Monitored progressive resistance training	No	Yes	3 weeks, 1.5 or 3 grams per day HMB-Ca	No	TOBEC for total FFM and FM	Strength: Average weight lifted during last 3 working sets of upper and lower body exercises	FFM: + 0.6 % FM: No Effect Strength: +2.6 to 17.4 % depending on lift
Jowko 2001 [10]	Active, college-aged males	Monitored progressive resistance training	No	No	3 weeks, 3 grams per day HMB-Ca	20 grams creatine per day for 7 days followed by 10 grams per day for 14 days	BIA	Strength: Cumulative 1-RM of major lifts (Squat, Bench Press, Clean)	FFM: + 0.6 % FM: - 0.7 % Strength: + 9 %
Kreider 1999[15]	Resistance trained, college-aged males males with > 1 year experience	Not monitored: Instructed not to change current individualized training regimens	No	No	28 days, 3 or 6 grams per day HMB-Ca	No	DXA for: LBM and FM	Strength: Bench Press and Leg Press	LBM: No Effect FM: No Effect Strength: No Effect
Gallagher 2000[12]	Untrained college-aged males	Monitored progressive resistance training	No	No	8 weeks, 3 or 6 grams per day HMB-Ca	No	7 site Skin Fold	Isometric and Isokinetic testing, Non-specific to training stimulus	FFM: + 3 % FM: - 1.6 % Strength: +2-3.5 % No differences between 3 and 6 g
Panton 2000[20]	Men and women, divided into untrained and resistance trained (> 6 months), 20–40 yrs of age	Monitored high intensity progressive resistance training	No	No	4 weeks, 3 grams per day HMB-Ca	No	Underwater Weighing	Bench Press and Leg Press 1-RM	FFM: +.5 kg FM: - .6 % Strength: +3-15 %
Hoffman 2004[19]	College Football players	Football camp, not controlled by investigators	No	No	10 days, 3 grams per day HMB-Ca	No	Not Measured	Wingate Power	No Effects
Kraemer 2009[13]	Recreationally active, college-aged males	periodized resistance training split	Yes	Yes	12 weeks, 3 grams per day HMB-Ca	14 grams arginine and 14 grams glutamine per day	DXA for LBM and FM and Limb Circumference	Squat and Bench Press 1RM Vertical Jump	LBM: + 40% FM: -40 % Strength: 50 % Power: +85 %
Thomson 2009[22]	Trained college-aged males	Non Monitored Assigned progressive resistance training program with 84 % compliance	No	No	9 weeks, 3 grams per day HMB-Ca	No	BIA	Bench Press, Preacher Curl, and Leg Extension 1-RM	FFM: 0.4 FM: - 3.8 Strength: 1.1-9.0 depending on lift
Portal 2011[23]	Elite adolescent volleyball players 13.5-18 yrs of age	Combination of progressive, resistance, and endurance exercise	Not reported	No	7 weeks, 3 grams per day HMB-Ca	No	DXA	Power on Wingate Strength of Bench Press and Leg Press	Fat: PL = +3.5% Vs. HMB= −6.6% FFM: PL= no change Vs. HMB= +3.7% Power: PL = +3% HMB = +13.5% Strength: PL=0-6.7 % vs. HMB +15.7 % - 23.5 %
Ransone 2003[24]	College football players	Progressive resistance and endurance exercise	No	No	4 weeks, 3 grams per day HMB-Ca	No	Skin Folds	Bench Press, Power Cleans, Squats 1-RM	FFM: +0.3 FM: - 3.8 Strength: 1.7 % increase
Kreider 2000 [18]	Trained, college football players	Offseason strength and conditioning program	Yes	No	4 weeks, 3 grams per day HMB-Ca	No	DXA	Bench Press, Power Cleans, Squats 1-RM, 12x6 second sprint performance	No Effects
O’Connor 2007[25]	Trained rugby players, 25 yrs of age	Progressive resistance training	No	No	6 weeks, 3 grams of HMB-Ca or HMB-Ca + Creatine per day	3 grams creatine per day	Skin Folds	Squat, Bench Press, and Deadlift 1-RM Wingate Power	Neither HMB-Ca nor creatine had an effect
Slater 2001[26]	College-aged, trained polo players and rowers	Non-controlled workouts assigned by the athletes’ respective coaches	Unknown	No	6 weeks, 3 grams per day HMB-Ca	No	DA	Bench Press, Hip Sled, Pullups 3-RM	No significant effects

* Abbreviations used in the table. TOBEC-total-body electrical conductivity; DXA-Dual-energy x-ray absorptiometry; BIA-bioelectrical impedance; FFM-fat free mass; FM-fat mass; LBM-lean body mass (TOBEC).

### HMB metabolism, pharmacokinetics and retention

#### Metabolism

**Figure 1:**
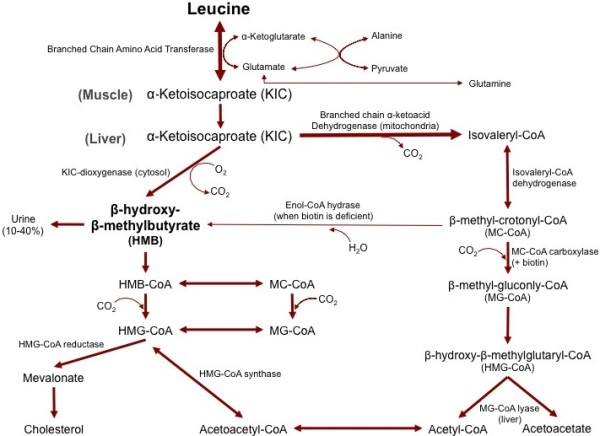
The metabolism of beta-hyroxy-beta-methyl-butyrate.

HMB is naturally produced in animals and humans from the amino acid leucine [27]. The first step in production of HMB is the reversible transamination of leucine to α-keto-isocaproate (KIC) by the enzyme branched chain amino acid transferase [28] (Figure 1). After leucine is metabolized to KIC, KIC is either metabolized into isovaleryl-CoA by the enzymeα-ketoacid dehydrogenase in the mitochondria, or into HMB in the cytosol, by the enzymeα-ketoisocaproate dioxygenase [28]. KIC is primarily metabolized into isovaleryl-CoA, with only approximately 5% of leucine being converted into HMB [28]. To put this into perspective, an individual would need to consume over 600 g of high quality protein to obtain the amount of leucine (60 grams) necessary to produce the typical 3 g daily dosage of HMB used in human studies [9]. Since consumption of this amount of protein is impractical, HMB is typically increased via dietary supplementation.

#### Rate of appearance and retention between varying forms of HMB

As a dietary supplement, HMB has been commercially available as a mono-hydrated calcium salt, with the empirical formula Ca (HMB)_2_-H_2_O (HMB-Ca). The magnitude and rate of appearance of HMB following ingestion is dependent on the dose, and whether or not it is consumed with additional nutrients. Specifically, Vukovich et al. [29] found that 1 g of HMB-Ca resulted in a peak HMB level in blood two hours following ingestion, while 3 g resulted in peak HMB levels 60 minutes after ingestion at 300% greater plasma concentrations (487 vs. 120 nmol·ml^-1^), and greater losses in urine (28% vs. 14%), for 3 and 1 g HMB-Ca ingestion, respectively. Peak HMB concentrations were also delayed by an hour and significantly lower (352 nmol·ml^-1^) when the HMB-Ca dosage was combined with 75 g of glucose. It is likely that the addition of glucose slowed gastric emptying, or improved HMB clearance. Recently a new delivery method of HMB, administered as a free acid, has been investigated [30]. The free acid form is called beta-hydroxy-beta-methylbutyric acid and can be designated as HMB-free acid (HMB-FA). The initial research studies have utilized HMB-FA associated with a gel, containing a buffering mechanism (K_2_CO_3_) that raises the pH to 4.5.

**Figure 2:**
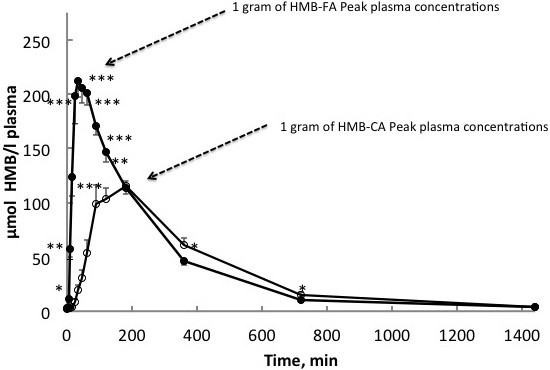
Absorbtion kinetics following ingestion of either 1 gram of calcium or free acid forms of HMB.

Commercially, HMB has only been available in the calcium salt form (HMB-Ca) as a powder, which has generally been supplemented in capsule form. Moreover, it was previously thought that because calcium dissociated relatively easily from HMB-Ca (10–15 minutes in the gut), there would be no difference in digestion kinetics between HMB-Ca and HMB-FA [31]. However, this is not the case as comparison of 0.8 g of HMB-FA to 1.0 g HMB-Ca (equivalent amounts of HMB) resulted in a doubling of peak plasma levels in one-fourth the time (30 vs. 120 minutes) in the HMB-FA compared with the HMB-Ca [30] (Figure 2). Moreover, area under the curve analysis of HMB concentrations over 180 minutes following ingestion was 91-97% greater in the HMB-FA than the HMB-Ca form. The half-life of HMB in plasma when given as HMB-FA and HMB-Ca were found to be approximately three- and two and a half hours, respectively [30]. Interestingly, even with greater peak plasma concentrations of HMB, urinary losses were not different between the two HMB forms. Perhaps the most intriguing findings were that plasma clearance, indicative of tissue uptake and utilization, was 25% greater with HMB-FA consumption compared with an equivalent HMB-CA consumption. To date, however, the majority of studies have been conducted using HMB-Ca.

#### HMB safety

The safety of HMB has been widely studied [32-36]. In a study conducted in compliance with Food and Drug Administration Good Laboratory Practice, rats consuming a diet of up to 5% HMB-CA for 91 days did not exhibit any adverse effects *vis a vis* clinical observations, hematology, clinical chemistry or organ weights [36]. This study reported no observed adverse effect levels (NOAEL) of 3.49 and 4.16 g·kg·BM^-1^ for male and female rats, respectively [36]. This would be the equivalent of an 81 kg human male consuming almost 50 g HMB-Ca per day for three months with no adverse effects, based on human equivalent dosing (HED) normalized to body surface area. In humans, consumption of 6 g HMB·d^-1^ for one month had no effect on cholesterol, hemoglobin, white blood cells, blood glucose, liver or kidney function [33]. In addition, two meta-analyses, one with HMB supplementation alone and another with HMB supplementation combined with glutamine and arginine, have concluded that HMB is safe and does not result in any adverse effects [34,35]. Moreover, Baier et al. [37] examined the effects of a 2–3 g of a daily ingestion of HMB-Ca in combination with amino acids for one year in the elderly and found that HMB consumption did not result in any changes in blood or urine markers of hepatic or renal function or blood lipids. Although the previous studies found no adverse events associated with HMB supplementation, a recent rodent study found an increase in plasma insulin after 320 mg·kg·BM^-1^/·d^-1^ supplementation for one month, which showed a significant increase in fasting insulin levels, suggesting a possible decrease in insulin sensitivity [38]. However, this finding has not been reported in any previous human study. Evidence to date indicates that that consumption of HMB is safe in both young and old populations; however, future studies examining the effects of HMB on insulin sensitivity in humans are warranted.

### The effects of HMB supplementation on skeletal muscle damage, protein breakdown, and recovery

HMB is presently thought to work by speeding regenerative capacity of skeletal muscle following high intensity or prolonged exercise [7]. Researchers have used a number of dependent measures to examine this attribute including serum indices of skeletal muscle damage (creatine kinase [CK], and lactate dehydrogenase [LDH]), and urinary indicators of protein breakdown (3-methyl-histidine [3-MH] and urea nitrogen) [10,11,17]. Perceived recovery and skeletal muscle soreness have also been investigated following training with, and without HMB supplementation [39]. Of the studies reviewed which investigated skeletal muscle damage and recovery (Table 1), there were a variety of supplement protocols (1 day to 6 weeks; pre vs. post exercise), age ranges (19–50 yrs), training protocols (progressive resistance vs. isokinetic dynamometer), and subject-training statuses (untrained, moderately to highly resistance trained, and endurance trained). Some studies included other supplements, such as creatine monohydrate, while others consisted of HMB alone. Diet and training were controlled in some studies, but not in others (Table 1). For these reasons, results across studies have not been consistent.

#### Effects of training status

Training status has been a variable that has received a great deal of interest in the literature. When training and/or diet are controlled, a number of studies have demonstrated that HMB can lower indices of skeletal muscle damage and protein breakdown in a dose dependent fashion in untrained populations [7,10,20]. For example, Nissen et al. [7] found that HMB blunted the rise in indicators of skeletal muscle damage and protein degradation, CK, LDH, blood and urinary urea nitrogen, and 3-MH (20-60%) after three weeks of high intensity, monitored resistance exercise. However, research indicates that in trained populations it is critical that the exercise stimulus is of adequate intensity and volume to cause skeletal muscle damage [40]. If these conditions are lacking, HMB is not likely to be efficacious [9]. Kreider et al. [15] examined the effect of HMB-Ca supplementation for 28 days in resistance-trained athletes. The training protocol of this study may have affected the outcome measures of this study. Participants were instructed not to change their training intensity or volume, thus no overload throughout the duration of the study occurred. As a result, no effect of the training or HMB-Ca was observed on indices of damage. This study was the first to indicate that HMB’s effects likely interact with both the exercise stimulus and the training status of the athlete. Similarly, Kreider et al. [18] also observed no changes in muscle catabolism after 4 weeks of HMB supplementation during a 28 day offseason conditioning program in Division 1 football players. Panton et al. [20] followed up with a large cohort of 41 subjects of untrained and moderately trained subjects (> 6 months of resistance training experience). They found that HMB-Ca blunted the rise in CK levels independent of training status during a monitored, high intensity progressive resistance-training program. Knitter and colleagues [11] also found that HMB decreased skeletal muscle damage after a 20 km run in well-trained runners (> 48 km per week) as indicated by decreased CK and LDH levels after the run. Recently, Wilson et al. [41] investigated the effects of pre exercise administration of HMB-FA to resistance trained athletes on muscle damage, and perceived recovery following a high volume resistance training bout centered around squats, deadlifts, and bench press. They found that HMB-FA supplementation blunted the rise in CK levels and protein breakdown following a workout compared to the placebo group. Moreover, HMB-FA improved the perceived recovery score, suggesting that HMB-FA enhanced recovery.

### Duration of supplementation, dose, and timing

The duration, dosage, and timing of HMB supplementation have notably varied in the literature (Table 1). The first study to look at the duration and dose of HMB was conducted by Nissen and colleagues [7]. Their results indicated that HMB-Ca attenuated protein breakdown to a greater extent following two weeks of supplementation than following one week, and that HMB-Ca was not able to significantly reduce CK concentrations until the third week of training. These effects appeared to be greater when ingesting 3 g of HMB-CA compared to lower doses of the supplement (1.5 g of HMB-CA). Other investigations who have supplemented with HMB-Ca for two or more weeks have generally reported that the supplement lowers indices of skeletal muscle damage and soreness, while those supplementing for shorter periods of time have not (Table 1). These findings suggest that HMB-Ca supplementation may be optimized when ingestion begins two weeks prior to the onset of a new training period or change in training workload.

In the majority of studies, however, researchers have had subjects consume HMB-Ca with breakfast, lunch, and dinner, without any regard to how the supplement is timed relative to exercise. Currently only two studies have reported HMB’s acute effects on skeletal muscle damage and recovery. Wilson et al. [17] examined the acute and timing effects of an oral 3 g bolus of HMB-Ca supplement on 16 untrained males using a unilateral, isokinetic leg extension based training protocol. These researchers found that HMB-Ca consumed 60 minutes prior to exercise prevented a significant rise in LDH, and tended to decrease soreness of the quadriceps relative to either the HMB-Ca supplement consumed following exercise, or a placebo supplement given prior to exercise.

Collectively these findings lead us to suggest the following: HMB supplementation appears to speed recovery in untrained and trained individuals if the exercise stimulus is high intensity, and/or high volume in nature. For untrained individuals this would likely occur with the introduction of most exercise regimens; however, in a trained population the exercise stimulus will likely need to center on free weights and compound movements. In regards to optimizing HMB supplementation, it appears that HMB has both acute and chronic effects. HMB’s acute effects likely depend upon supplementation pre-exercise. If taking HMB-Ca, the recommendation would be to consume 3 g, at least 60 minutes prior to intense exercise. If consumed with glucose it may need to be taken as long as two hours prior to training. HMB in the HMB-FA form may have an overall faster and greater effect based upon the rise in plasma levels. Thus, athletes could consume the supplement in HMB-FA form 30–60 minutes prior to exercise. Finally, in order to optimize HMB’s chronic effects, the recommendation would be to consume 3 g daily, divided into three equal servings for a minimum of two weeks prior to a potentially damaging skeletal muscle event.

### The effects of HMB supplementation on skeletal muscle hypertrophy in healthy untrained and trained adults

HMB’s effects on skeletal muscle mass, strength, and hypertrophy have been studied in exercising humans for nearly two decades [7,9]. Similar to its reported effects on skeletal muscle damage, a wide range of subject populations (untrained vs. resistance trained; male vs. female) and training protocols (Table 2) have been examined. Training protocols have varied in duration (10 days to 12 weeks) [13,19], periodization scheme [13,42]), and training modalities (machines and free weights [22] vs. free weights only [42]) (Table 2). To confound the situation further, some researchers have designed and monitored the resistance-training protocol [7,13,20], while others have left it up to subjects to train on their own [15,22]. In other cases, subjects have participated in unspecified training protocols reportedly provided by various team coaches or training camps [19,26]. In addition, studies have provided a variety HMB doses ranging from 1.5 to 6 g daily [7,12]. Moreover, some studies have supplemented HMB along with creatine monohydrate [10,43] or arginine and glutamine [13]. Further, some researchers have controlled for diet [13,42], while the majority have not [10,12,19,22,34]. Lastly, the outcome measures for indices of skeletal muscle mass have varied from less accurate indirect indices (skin fold and bioelectrical impedance measures) [10,12,22], to dual x-ray absorptiometry (DXA) [13] to determine fat free mass (FFM) and LBM, respectively. Thus, in order to make any overall conclusions on HMB’s effectiveness, the validity and reliability of each of these measures needs to be considered.

### Training status and its interaction with variation of training load and duration of training protocol

#### Untrained individuals

In both trained and untrained individuals the majority of studies using HMB have lasted four weeks or less (Table 2). In untrained individuals supplementation with HMB has been demonstrated to increase FFM, as well as strength in as little as three weeks [7,10]. These findings are not surprising if HMB operates through speeding recovery of damaged skeletal muscle tissue [7,10,20]. In particular, research indicates that the initial weeks of training result in the highest magnitude of damage in an untrained population [40,44] (Table 2). Research supports that rate of improvement in novice lifters decline as their training experience increases, [45], however, the majority of studies using HMB were not periodized. For these reasons HMB’s magnitude of effect over a placebo in novices only slightly increases when analyzing results over eight weeks [12] versus three to four weeks utilizing a linear resistance training model [7,10]. Finally, in untrained individuals it appears that 3 g of HMB·d^-1^ produces greater gains than 1.5 g of HMB·d^-1^[7]; though, 6 g of HMB·d^-1^ was not shown to further increase HMB’s effectiveness over 3 g of HMB·d^-1^[12]. However, only one study has examined a daily dose of 6 g HMB, therefore no definitive recommendation on (upper limit) dosing can be provided until additional research is conducted.

According to the available science, the effectiveness of HMB appears to be optimized under conditions of continually changing loading patterns [9]. Specifically, Kraemer and colleagues [13] had recreationally active, but not resistance-trained, individuals participate in a 12-week, periodized training program. Subjects were randomly assigned to 3 g daily of an HMB-Ca supplement that contained 14 g glutamine and 14 g arginine, or a placebo in a double-blinded manner. The training program consisted of three constantly changing loading patterns targeting a strength, hypertrophy, and strength endurance continuum. Moreover, these researchers controlled for subjects’ diets, and monitored every training session. Results showed that these previously untrained subjects in the HMB-Ca group experienced greater gains in LBM (+ 3.5 kg in placebo vs. + 9 kg HMB-Ca), and squat strength (+29 kg in placebo vs. + 46 kg in HMB-Ca ).

#### Trained individuals

The rate of adaptation in strength, power, and hypertrophy in trained and untrained individuals markedly differs. For example Ahahtanin et al. [46] found that 21 weeks of resistance training resulted in 21% and 4% increases in strength in untrained and highly strength trained athletes, respectively. In these subjects, HMB appears to augment adaptations following unaccustomed high intensity training protocols. Because the rate of adaptation is markedly slowed in trained populations it is likely that HMB’s effects in this population will be optimized over longer duration protocols (>6 weeks). For example, the majority of studies in trained individuals lasting six weeks or less found little to no significant differences with HMB-Ca compared to a placebo [15,18,19,26]. However, those lasting longer than six weeks generally elicited positive effects in strength, and FFM [7,22,42].

The capacity of a training protocol to provide a novel training stimulus may be critical to consider when studying HMB. To date, the majority of studies have been linear in nature, and not monitored by the investigator (Table 2). The first study conducted in trained individuals lasted 28 days, and subjects were instructed to maintain their normal training protocols [15]. Neither the placebo nor HMB-Ca supplementation resulted in increases in CK or strength, thus suggesting that HMB may not work without a novel training stimulus. Following this study, Slater et al. [26] recruited trained water polo and rowing athletes. For this study the training protocol lasted six weeks, and again was not controlled by the investigators; however, the athletes were under the supervision of their respective strength coaches. As such, subdivisions of athletes in this protocol each experienced variable training stimuli making it extremely difficult to determine any direct effects of HMB supplementation. For this reason, no effects of HMB-Ca were noted.

The most recent study using HMB-Ca was conducted by Thomson and colleagues [22]. These researchers supplemented individuals with reportedly one year or more of resistance training experience with 3 g of HMB-Ca or a placebo while performing a linear (periodized) resistance-training program. Subjects were asked to follow the program for nine weeks; however, they were not monitored. Subject compliance to the training program was on average 84 ± 22%. These last two points are critical to analyze for two reasons. First, a 20% lack of compliance lowers overall training frequency, which decreases the probability of optimizing HMB’s effects on recovery rate. Second, research demonstrates that directly supervised, heavy-resistance training results in a greater rate and magnitude of training load increases in resistance-trained individuals[47]. Moreover, supervised training results in greater maximal strength gains compared with unsupervised training [48]. For this reason it is likely that the training stimulus and frequency in this nine week study did not exploit HMB’s capacity to speed recovery under maximal, and constantly varying training stimuli. An additional confounding variable in this study was that skeletal muscle hypertrophy (FFM) was estimated from bioelectrical impedance, which has been demonstrated to have high variability [49]. Finally, the outcome strength measures were single joint movements (e.g., biceps curl and leg extension). If HMB increases overall lean mass, it may have been more appropriate to select multi-joint, structural exercises such as the squat and/or bench press. However, even with these limitations nine weeks of HMB-Ca supplementation resulted in small, but statistically significant decreases in FM, and increases in FFM and strength.

To date, few studies have examined monitored resistance training in trained athletes [7,18,20,42]. Of these, only one exceeded six weeks in duration. The first was conducted by Kreider et al. [18] who examined the effects of four weeks of HMB supplementation during a supervised offseason strength and conditioning program in college football players and observed no changes in lean mass or strength. However, Panton et al. [20], examined the effects of four weeks of HMB supplementation during resistance training in 36 women and 39 men (20–40 yrs) with varying levels of training experience. Their training protocol consisted of very high intensity loads (>80 % 1-RM) which were consistently adjusted as subject tolerance for a given weight increased. Due to the high intensity nature of the protocol, the HMB-Ca group showed greater decreases in body fat compared with placebo supplementation (−1.1 % vs. -0.5%, respectively); increases in bench press strength (7.5 kg vs. 5.2 kg, respectively); and LBM (1.4 kg vs.0.9 kg, respectively). These changes were independent of training experience. Moreover, Nissen et al. [7] conducted a seven week high intensity (>80% 1-RM) training study in individuals who could bench press ≥ 135 kg and squat greater than 1.5 times their bodyweight and found that subjects supplemented with HMB-Ca gained an average of 4.5 kg more on their bench press and 3.2 kg more on their squat when compared to the placebo supplemented subjects.

Collectively the findings presented in Table 2 lead us to the following conclusions: 1) in untrained individuals, HMB can enhance muscle hypertrophy and dynamic strength in as little as three weeks; however, 2) for trained individuals it is important to realize that adaptations occur at a slower rate than in untrained individuals [46]. For this reason, HMB will likely be most beneficial over longer training durations (> 6 weeks) in trained individuals. HMB supplementation has been demonstrated to result in modest increases in strength during unsupervised, resistance training programs greater than six weeks in duration. Presently, available literature suggests 38 mg·kg·BM^-1^daily, divided into two to three servings provides an adequate amount of HMB to enhance adaptive processes in muscle. However this prescription is far from refined, as no research has investigated the optimal dosage of HMB per serving to optimize protein balance. Research has also not focused on the ideal distribution (e.g. number of times HMB should be consumed per day) needed to optimize HMB’s effects. Finally, more research needs to be done comparing HMB-FA to HMB-Ca. Supplementation with HMB-FA has been shown to increase HMB levels to a greater and more rapid peak in blood than supplementation with HMB-Ca. The HMB is also retained to a greater extent as well. It is plausible that these differences may augment the effects of HMB-Fa on overall adaptive processes.

### HMB in athletes training in an energy restricted state

The effects of HMB supplementation on regenerative capacity and fat metabolism make it a unique candidate for a number of special situations in which skeletal muscle wasting is indicated. One situation in particular concerns caloric (energy) restriction. Restricting calories prior to competition is commonly used by bodybuilders and those in weight-classified sports. However, research demonstrates that calorie restriction can cause decreases in lean mass and exercise performance [50]. In a recent study [50] on female judo athletes who were calorically restricted for three days, body weight and body fat percentage were significantly decreased in the subjects consuming HMB-Ca compared to the control group. There were also trends for HMB to have positive effects on LBM, which tended to decrease more in the control group (−1.6%) than in the HMB group (−0.5%). Peak power decreased by nearly 11% in the control group compared to only 5% in the HMB group. These findings suggest that individuals who are moderately calorically restricted may augment fat loss and prevent declines in LBM by supplementing with HMB.

### HMB supplementation in youth and adolescent populations

Research in infants using HMB has yet to be done using human models. However, there is recent epigenetic data in animal models to suggest that HMB given during pregnancy can result in prenatal programming of skeletal muscle tissue. Specifically, maternal supplementation of HMB during pregnancy resulted in greater weight and lean mass gain in piglets than those not under maternal treatment [51]. Moreover, research in growing, pre-adolescent rats suggests that HMB supplementation was able to stimulate skeletal muscle hypertrophy in the extensor digitorum longus and soleus muscles [52], and that HMB was able to increase the mTOR and phosphorylation of p70S6K in the EDL muscle [52].

There is very little research examining the effects of HMB in human adolescent populations. However, this population may be an ideal model for HMB supplementation as resources required to augment their training adaptations compete with resources needed for normal growth of organs, bones, and muscle tissue [53-55]. HMB assists in recovery in challenging situations, and therefore may be beneficial to younger populations. In a recent study the effects of 3 grams per day of HMB-Ca on male and female elite adolescent (13–18 yrs) volleyball players during the first seven weeks of their training season was investigated [56]. Their results demonstrated that FFM increased in the HMB-Ca supplemented group, but not placebo supplemented group. Moreover FM declined (−6.6 %) in the HMB-Ca supplemented, but not placebo supplemented group (+3.5 %). In addition, Wingate test peak power, and upper- and lower-body strength were greater with HMB-Ca supplementation. No changes in hormone status (testosterone, cortisol, IGF-1, growth hormone) or inflammatory mediators (IL-6 and IL-1 receptor antagonist) occurred with HMB-Ca supplementation.

### HMB supplementation in aging and masters athletes

Skeletal muscle loss is a part of the aging process and approximately 30% of skeletal muscle mass is lost between the 5^th^ and 8^th^ decades of life [57]. This reduction in skeletal muscle mass occurs for several reasons, including maintaining a sedentary lifestyle, malnutrition, insulin resistance, oxidative stress, and alterations in skeletal muscle metabolism and repair (as reviewed by Kim et al. [58]). In addition, the elderly exhibit impaired anabolic and anti-catabolic responsiveness to resistance exercise and amino acid feeding, termed anabolic resistance [59]. Anabolic resistance can be overcome by supplementation of leucine, and it has been hypothesized that this may be due to the conversion of leucine to HMB [52]. These data suggest a potential benefit of HMB supplementation in aging individuals [58,60].

Studies have investigated the effects of nutritional supplements containing HMB, without an exercise intervention, on skeletal muscle mass in the elderly (reviewed by [61]). Flakoll et al. [62] investigated the effects of 12 weeks of either HMB, arginine and lysine supplementation or placebo supplementation in 50 elderly subjects and observed an increase in LBM, leg strength, handgrip strength, and a decreased “timed up and go” test time in the HMB-supplemented group compared to the placebo-supplemented group. Baier et al. [37] investigated the effects of one year of either HMB, arginine, and lysine supplementation or control supplementation in 77 elderly subjects over 65 years of age and observed significant increases in lean mass in the HMB-supplemented group and no change in lean mass in the control-supplemented group. Moreover, an increased rate of protein turnover in the HMB group and a decreased rate of protein turnover in the placebo group were observed after both three and 12 months of supplementation. In addition to the beneficial effects of HMB on skeletal muscle, HMB supplementation may also have effects on body fat. Wilson et al. [63] investigated the effects of 16 weeks of HMB supplementation in aged rats and found that body fat mass, as measured by DXA, increased by nearly 50% from young to middle age, and that HMB supplementation prevented this gain in body fat with aging. Moreover, these researchers also found that HMB supplementation was able to prevent the loss of skeletal muscle fiber size in very old as compared to young rats. These studies suggest that HMB alone can decrease body fat and increase skeletal muscle mass and strength in aging populations.

The efficacy of HMB supplementation in conjunction with a strength-training program has also been investigated in aging populations. Vukovich et al. [64] compared the effects of eight weeks of either HMB or placebo supplementation on body composition and strength in 70 year old men and women performing a strength training program. A trend (p=0.08) towards an increase in lean mass was observed in the HMB-supplemented group, while no change was observed in the placebo-supplemented group. However, it should be noted that body composition was measured with skinfold calipers in this study. The HMB-supplemented group also had an approximate 8% decrease in fat mass. Upper and lower body strength increased by 15-20%; however, there was no difference in strength changes between the groups. While the differences observed were not statistically different with HMB supplementation, it should be noted that the training protocol in this study consisted of 2 sets of 8–12 repetitions 2 days per week. Thus, this particular study suggests that in previously untrained older adults the use of HMB may not provide any further benefit than training alone. Considering the paucity of available research on HMB ingestion and resistance exercise in older adults, additional investigations are warranted.

### HMB improves indices of aerobic performance, fat loss, and energy metabolism

While HMB has long been touted as an anti-catabolic agent that may aid recovery and improve performance, recent evidence has identified additional metabolic benefits of HMB supplementation related to energy metabolism. This section will discuss how HMB may improve aerobic performance as well as increase fat loss and mitochondrial biogenesis, and the purported mechanisms of action underlying these benefits.

Previous studies have demonstrated the potential benefits of HMB for aerobic athletes. For instance, Vukovich and Dreifort [65] investigated the effects of HMB supplementation on peak oxygen consumption (VO_2_peak) and the onset of blood lactate accumulation (OBLA) in eight endurance-trained master-level competitive cyclists having an average training volume of 300 miles per week. Participants performed a graded cycle ergometer test until exhaustion. All participants performed three 2-week supplementation protocols consisting of either 3 g of HMB-Ca, 3 g of leucine, or a placebo daily, while continuing their normal training volume. Results from the graded exercise test indicated that HMB supplementation increased the time to reach VO_2_peak (8%), while leucine and the placebo did not. The VO_2_ at 2 mM blood lactate (OBLA) increased with HMB (9.1%) and leucine (2.1%) supplementation, but did not change with placebo supplementation.

The mechanisms for these benefits of HMB on aerobic performance and fat loss are poorly understood. However, recent evidence demonstrated that HMB supplementation improves fatty acid oxidation, adenosine monophosphate kinase (AMPK), Sirt1 (Silent information regulator transcripts) and Sirt3 activity in 3T3-L1 adipocytes and in skeletal muscle cells [66]. To elaborate, the Sirt proteins belong to a class of NAD+− dependent protein deacetylases involved in energy metabolism, which sense energy balance through changes in the NAD+/NADH ratio. Sirt proteins modify the acetylation level of histones and proteins [67]. Adenosine mono-phosphate protein kinase (AMPK) is also a sensor of energy balance, but does so through changes in AMP/ATP ratios [68]. Collectively, these proteins act to improve mitochondrial biogenesis, fat oxidation, energy metabolism, and the reactive oxygen defense system [67-69]. Consequently, this recent evidence has shown that HMB supplementation increases mitochondrial biogenesis and fat oxidation [70].

Exactly how HMB induces changes in Sirt proteins, AMPK, and mitochondria remains unclear. However, these results could have implications for obesity, insulin resistance, and diabetes, as well as for athletes seeking to improve body composition and aerobic performance.

### Proposed mechanisms of action

**Figure 3:**
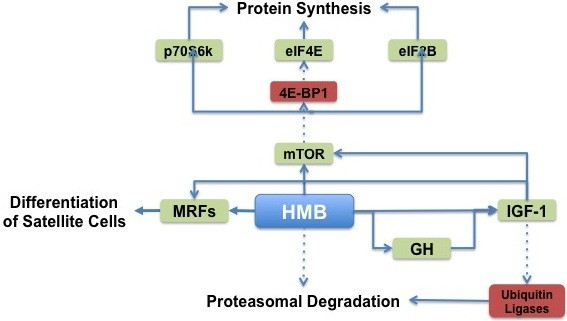
HMB’s proposed mechanisms of action.

Skeletal muscle protein turnover is the product of skeletal muscle protein synthesis and skeletal muscle protein degradation [71]. When protein synthesis exceeds protein degradation, there is a net synthesis of skeletal muscle protein. However, when protein degradation exceeds protein synthesis, there is a net breakdown of skeletal muscle protein. HMB has been shown to affect both protein synthesis and degradation pathways in skeletal muscle and the effect of HMB on these pathways is summarized below and in Figure 3.

#### Protein synthesis

HMB has been shown to stimulate protein synthesis in skeletal muscle [72]. This has been hypothesized to occur through stimulation of mTOR, a protein kinase responsive to mechanical, hormonal, and nutritional stimuli. Mammalian target of rapamycin has a central role in the control of cell growth, primarily by controlling mRNA translation efficiency [6]. Indeed, previous studies have observed that HMB supplementation increases phosphorylation of mTOR and its downstream targets ribosomal protein S6 kinase (S6K) and eukaryotic initiation factor-4 binding protein-1 (4EBP1) [73,74].

The growth hormone (GH) and insulin-like growth factor 1 (IGF-1) axis may also play a key role in the stimulation of protein synthesis, and it is possible HMB may stimulate protein synthesis through changes in the activity of GH/IGF-1 axis. Gerlinger-Romero et al. [75] observed an increase in pituitary GH mRNA and protein expression after one month of HMB supplementation. Moreover, liver IGF-1 mRNA and serum IGF protein levels were also increased in the HMB-supplemented rats; however, this occurred without an increase in skeletal muscle IGF-1. In contrast, an increase in skeletal muscle insulin-like growth factor-1 (IGF-1) has been observed after HMB treatment of chicken and human myoblasts [76]. Taken together, these results suggest that HMB may affect GH/IGF-1 axis signaling; however, the effect on skeletal muscle protein synthesis requires more investigation. It is possible that the GH/IGF-1 axis signaling may require a large change in plasma HMB levels. At this point, it is not clear whether a threshold response to a specific concentration of plasma HMB exists. This certainly merits further investigation.

#### Skeletal muscle regeneration

In addition to the direct effects on protein synthesis, HMB has been shown to affect satellite cells in skeletal muscle. Kornaiso et al. [76] cultured myoblasts in a serum-starved state to induce apoptosis. When myoblasts were cultured with HMB, the mRNA expression of myogenic regulatory factor D (MyoD), a marker of cell proliferation, was increased in a dose responsive manner. Moreover, the addition of various concentrations of HMB (25–100 μg/ml) to the culture medium for 24 hours resulted in a marked increase of myogenin and myocyte enhancer factor-2 (MEF2) expression, markers of cell differentiation. As a result, there was a significant increase in the number of cells, suggesting a direct action of HMB upon the proliferation and differentiation of myoblasts.

#### Skeletal muscle proteolysis

Skeletal muscle proteolysis is increased in catabolic states such as fasting, immobilization, aging, and disease [77]. HMB has been shown to decrease skeletal muscle protein degradation both *in vitro*[72,73] and *in vivo*[78]. The mechanisms whereby HMB affects skeletal muscle protein degradation are described below.

The ubiquitin-proteasome system is an energy-dependent proteolytic system that degrades intracellular proteins. The activity of this pathway is significantly increased in conditions of exacerbated skeletal muscle catabolism, such as fasting, immobilization, bed rest and disease [77]. Therefore, inhibition of this proteolytic system could explain the attenuation of skeletal muscle protein losses observed during treatment with HMB. Indeed, HMB has been shown to decrease proteasome expression [72] and activity [72,78-80] during catabolic states, thus attenuating skeletal muscle protein degradation through the ubiquitin-proteasome pathway.

Caspase proteases induce skeletal muscle proteolysis through apoptosis of myonuclei and are commonly up-regulated in catabolic states. However, HMB has also been shown to attenuate the up-regulation of caspases, reduce myonuclear apoptosis in catabolic states, such as skeletal muscle cells cultured with large concentrations of inflammatory cytokines [81], and skeletal muscle unloading [82]. Thus, in addition to its effects on the ubiquitin-proteasome pathway, HMB may also attenuate skeletal muscle protein degradation through inhibition of caspase activity.

#### Summary of mechanisms

HMB has been shown to result in a net positive balance of skeletal muscle protein turnover though stimulation of protein synthesis and attenuation of protein degradation. HMB induces protein synthesis through up-regulation of the mTOR pathway while HMB attenuates protein degradation through attenuation of the ubiquitin-proteasome pathway and caspase activity. Moreover, HMB stimulates skeletal muscle satellite cell activation and potentially increases skeletal muscle regenerative capacity.

## Conclusions

High intensity resistance training is essential for athletes seeking to add strength and hypertrophy. However, high intensity resistance training that results in skeletal muscle damage may take a number of days to recover from; in this case, overall training frequency may be reduced. HMB appears to speed recovery from high intensity exercise. These effects on skeletal muscle damage appear to be reliant on the timing of HMB relative to exercise, the form of HMB, the length of time HMB was supplemented prior to exercise, the dosage taken, as well as the training status of the population of interest. In particular, the supplement should be taken at 1–2 grams 30–60 minutes prior to exercise if consuming HMB-FA, and 60–120 minutes prior to exercise if consuming HMB-Ca. Finally, it is likely that HMB will work ideally if consumed at a dosage of 3 grams for two weeks prior to a high intensity bout that induces muscle damage.

HMB appears to interact with the training protocol utilized, as well as the experience of the athlete. In untrained individuals, low volume, high intensity resistance training will cause enough skeletal muscle tissue disruption to benefit from HMB supplementation. In addition to speeding recovery from high intensity exercise, HMB may assist athletes in preventing loss of lean body mass in catabolic situations such as caloric restriction. HMB may also be beneficial for augmenting body composition and physical performance in master’s level athletes, or aging individuals in general. Finally, although research is limited it appears that the supplement may also enhance aerobic performance.

## Competing interests

JMW has received external grants from industry to affiliated institutions to conduct exercise and nutrition research. PJF has no competing interests to declare. BC has received university and private sector funded grants to conduct research on several dietary supplements and has received compensation for speaking at conferences and writing lay articles/books about dietary supplements. GJW has no competing interests to declare. NZ has no competing interests to declare. LT has received academic and industry funding related to dietary supplements and honoraria for speaking at conferences. CW has received external grants to conduct exercise and sport nutrition research. DK works for a Contract Research Organization that has received research grants from the pharmaceutical and nutrition industries. JRS is currently a science advisor to Abbott Nutrition. JRH currently conducts research for Metabolic Technologies Inc. TNZ has received external grants from industry to conduct nutrition and supplement research and is a science advisor for Biotest Labs LLC. HLL has received funding from industry to conduct clinical research through The Center for Applied Health Sciences, has consulted for multiple dietary supplement and medical food companies, and currently serves as scientific and medical advisor to Nordic Naturals, Inc. RK has received external grants from industry to affiliated institutions to conduct exercise and nutrition research, serves as a legal expert on exercise and nutrition related cases, and currently serves as a scientific advisor for Woodbolt International. AESR has received external grants from industry to affiliated institutions to conduct exercise and nutrition research. JA is a sports science consultant for VPX/Redline.

## Authors’ contributions

JMW prepared the draft of the position stand for review and editing by coauthors. The final draft was then reviewed and edited by all coauthors which was then reviewed, approved, and adopted as the official position of the ISSN by the Research Committee. All authors read and approved the final manuscript.
